# Highlights of the 2026 Pulmonary Vascular Research Institute Conference: Celebrating 20 Years of Progress and Innovation

**DOI:** 10.1002/pul2.70275

**Published:** 2026-05-08

**Authors:** Laura C. Price, Katarina Zeder, Sasha Z. Prisco

**Affiliations:** ^1^ National Pulmonary Hypertension Service, Royal Brompton Hospital part of GSTT Foundation Trust London UK; ^2^ National Heart and Lung Institute Imperial College London UK; ^3^ Institute for Health Computing University of Maryland North Bethesda Maryland USA; ^4^ Division of Pulmonary, Critical Care and Sleep Medicine University of Maryland School of Medicine Baltimore Maryland USA; ^5^ Cardiovascular Division University of Minnesota Minneapolis Minnesota USA

## Abstract

The 2026 Pulmonary Vascular Research Institute Conference was held in Dublin, Ireland, and celebrated 20 years of scientific achievements in pulmonary vascular disease, with focus on drug development milestones, translational advances, artificial intelligence, and precision medicine. Professional networking opportunities included the Networking Reception, Early Career Luncheon, and Women in Pulmonary Hypertension Luncheon.

Founded in 2006, the Pulmonary Vascular Research Institute (PVRI) recently gathered in Dublin for its annual conference, themed “20 Years of Progress and Innovation”, to honor two decades of scientific achievements in pulmonary vascular disease. The 2026 conference stood out by reviewing key milestones in pulmonary hypertension (PH) research while also establishing the aims and aspirations for the next 20 years and beyond. Fittingly, this year's conference had a record‐high number of registrations. A key highlight of this year's conference was the Innovative Drug Development Initiative (IDDI) satellite meeting. The IDDI meeting emphasized the differences between populations participating in randomized controlled trials and those encountered in clinical practice, highlighting that up to 40% of pulmonary arterial hypertension (PAH) patients would not be eligible to participate in these trials due to the restrictive inclusion and exclusion criteria [1]. Whether the inclusion criteria should be widened to include currently ineligible patients, particularly considering the changing demographics of current PAH patients (older age and more comorbidities), was a key question tackled in the IDDI meeting. Perspectives from clinicians, sponsors, and regulatory officials were discussed. Preceding the main conference, several additional satellite meetings were held by the Latin America, Imaging, Paediatric and Congenital, and High Altitude Task Forces as well as the Infection in Pulmonary Vascular Disease (iPVD) Consortium. The day after the main meeting, the International Consortium for Genetic Studies (PAH‐ICON) convened.

## 1 Basic and Translational Highlights

The main meeting opened with invigorating discussions on the clinical efficacy, hemodynamic and right ventricular (RV) effects, adverse events, and preclinical studies related to sotatercept. Now, two years after FDA approval in the United States, sotatercept has revolutionized PAH treatment. Preclinical and clinical studies suggest that sotatercept may impair RV contractility, a finding that requires further validation and could be concerning for non‐responders who do not experience a reduction in pulmonary vascular resistance. Preclinical efforts are underway to selectively target BMP9 to mitigate potential adverse effects of sotatercept/ACTRIIA‐Fc (an activin/GDF/BMP10 and mild BMP9 ligand trap), such as telangiectasias, epistaxis, and gastrointestinal bleeding.

The current PVRI President, Dr. Anna Hemnes, presented data on the role of mitochondria and metabolism in PH and RV dysfunction. Early studies exploring the potential effects of the lung basement membrane were also presented. Additionally, compelling data were shown on an inflammatory lung–vascular–brain axis linking PAH with cerebellar and frontal lobe cerebral alterations.

Other basic and translational highlights of the meeting included an overview of the power of leveraging multiple omics approaches to nominate potential new therapeutic targets; translation of complex DNA repair mechanisms and epigenetic targets into clinical trials; cellular senescence in PH; animal models and molecular targets for PH due to chronic obstructive pulmonary disease (COPD); and the effects of sleep and circadian rhythm disruption on PAH pathogenesis.

## 2 Clinical Highlights

Clinical highlights of the conference included inspiring keynote lectures focused on PH associated with chronic lung diseases and the important role of further analysis of negative trials (e.g. the PERFECT study [2], which a criteria for potential responders and non‐responders were identified in post hoc analyses [3]), the value of chest computed tomography (CT) scans to better phenotype lung diseases in future trials, and the hope for better therapies for PH‐COPD and PH‐interstitial lung disease (ILD), with several current Phase 2 trials testing novel anti‐remodeling pathways.

Other key highlights emphasized the clinical role of the right heart, including both the right atrium and RV, which remain underexplored in the progression of PH and RV dysfunction. Further, the global epidemiology and burden of PH were discussed for adults as well as the pediatric population, which is a key interest of the PVRI. Investigators of the PVRI GoDeep Registry highlighted the power of worldwide collaboration and real‐world data. Key emphasis was also laid on chronic thromboembolic PH (CTEPH) and chronic thromboembolic pulmonary disease (CTEPD), particularly on advances in balloon pulmonary angioplasty, as highlighted by experts from Japan [4] and Austria, as well as unresolved clinical questions, such as whether normalization of hemodynamics should be a treatment goal and whether all possible thromboembolic lesions should be resolved. Other keynote lectures discussed the clinical relevance and safety of wearables (particularly CardioMEMS [5]) for making treatment decisions not only for PAH but also for other PH groups.

Another notable highlight was the integration of adult and pediatric aspects of PH, offering a comprehensive view of disease mechanisms. This synergy was effectively illustrated by the discussions on RV function in congenital heart disease and Group 3 PH associated with bronchopulmonary dysplasia.

Rapid pro‐con debates resulted in audience interaction and emphasized the need for further evidence to solve certain clinical dilemmas, such as the treatment approaches for PH‐COPD and CTEPH. During the highly engaging “Sharks Den,” investigators pitched research approaches on novel PH biomarkers, targets, and devices that the audience then voted on. This year's winning presentation was on the potential of ex vivo PH models as a surrogate for rodent models.

## 3 Precision Medicine and Artificial Intelligence (AI)

The third day of the main conference was dedicated to advances in precision medicine and AI in PH. In PH, AI has been applied at different stages of the disease. As a screening tool, AI has been deployed for automated waveform analysis of electrocardiograms, as has been shown previously [6]. Another screening potential lies in AI‐driven analysis of imaging modalities. Here, segmentation and deep learning of cardiac magnetic resonance imaging (MRI), chest CT, and echocardiography [7] have shown great potential for improved prognostication and classification of PH patients [8], which may be particularly attractive for low‐resource areas.

In addition to these advances, the development of a clinical AI‐driven support tool that mines right heart catheterization (RHC) data was presented. Here, the development of an agentic large language model (LLM) showed very high precision and recall in structuring and extracting RHC variables from clinical reports. Further, machine learning was used to impute missing variables in RHC, which is an often‐encountered problem when working with data captured in the electronic health record [9].

The use of precision medicine to identify drug targets on a molecular level was also discussed. For example, liquid biopsies and the isolation of pulmonary arterial endothelial cells from balloon catheter tips during RHC procedures were combined with network analyses. Using this approach, the investigators aimed to identify individual drug receptors on the endothelial cells and match those to drugs approved for PAH therapy [10]. The goal of this approach is to increase personalized therapy choices for patients based on molecular fingerprints, thus a further step toward identifying the right therapy for the right patient in PH.

## 4 Networking at PVRI

**Figure 1:**
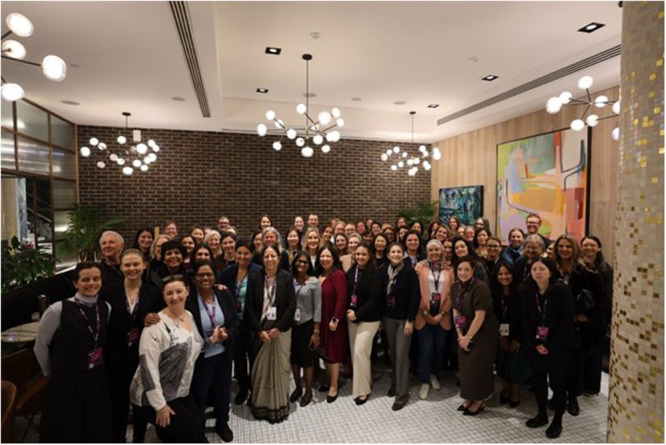
Women in PH Luncheon (Organizers: Katarina Zeder and Soni Savai Pullamsetti).

The PVRI conference stands out for the multiple opportunities to strengthen and broaden professional networks within the PH community and to welcome new and early‐career members. Among these events, the evening networking reception after the first day of the main meeting was a particular highlight. In addition, well‐attended poster sessions throughout each day of the main conference were perfect opportunities to engage with early and established researchers. Moreover, the Early Career Speed Mentoring Luncheon provided an ideal moment to connect young researchers with experts in the PH field. The Women's Luncheon provided space for women to connect and build strong professional relationships (Figure 1). A special tribute to our beloved and deeply missed colleague, Dr. Lucilla Piccari, a former highly active member of PVRI, was paid and will be remembered through future research collaborations.

The Gala Dinner was held in the legendary Guinness Storehouse, which included a tour of the brewery, a relaxed dinner, an awards ceremony, and subsequent time for socialization in an environment that included a live band and energetic singing and dancing. Dr. Stephen Archer was awarded the PVRI Lifetime Achievement Award; Drs. Sebastien Bonnet, Steeve Provencher, and Gabor Kovacs received the PVRI Achievement Awards; Sylvia Nikkho received the Certificate of Recognition; Dr. Daniel Colon Hidalgo earned the Butrous Foundation Young Investigator Award; and Drs. Katarina Zeder, Will Oldham, and Abdullah Aldalaan were awarded the PVRI Special Recognition Award. Fundraising events this year included an early morning walk/run tour of Dublin and a pub crawl.

**Figure 2:**
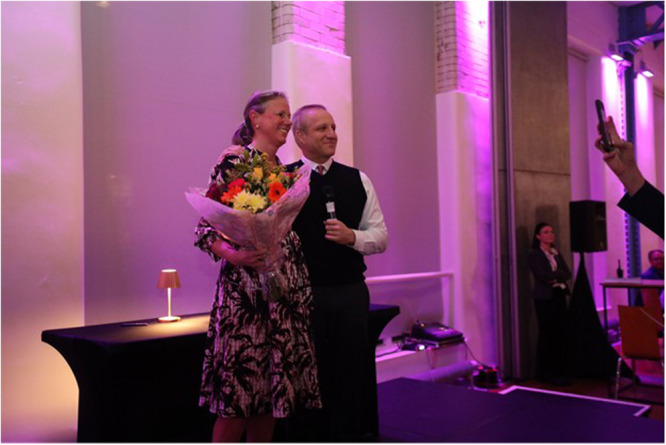
Outgoing PVRI president, Dr. Anna Hemnes with the incoming president, Dr. Bradley Maron.

The PVRI and the newly elected president, Dr. Bradley Maron (Figure 2), are looking forward to welcoming you to the 2027 PVRI Conference in Bangkok, Thailand!

## Author Contributions

All authors participated in drafting the original manuscript, critical revision of the manuscript, and approved the submission of the paper.

## Conflicts of Interest

Laura C. Price reports steering committee roles with Insmed Incorporated and Pulmovant; research support to her department from Ferrer, and funding from AOP Orphan Pharmaceuticals, Johnson & Johnson, Liquidia, and United Therapeutics. Katarina Zeder has grants from United Therapeutics and the Cardiovascular Medical Research and Education Fund (CMREF) (outside the scope of this work) and is a Consultant for AstraZeneca (outside the scope of this work). Sasha Z. Prisco is funded by an American Heart Association Career Development Award (23CDA1049093, https://doi.org/10.58275/AHA.23CDA1049093.pc.gr.167948) and by NIH K08 HL168166 and is on the Speakers Bureau for Merck.
